# Evaluating prognosis by CK7 differentiating renal cell carcinomas from oncocytomas can be used as a promising tool for optimizing diagnosis strategies

**DOI:** 10.18632/oncotarget.10225

**Published:** 2016-06-22

**Authors:** Fuling Ma, Liang Dai, Zhun Wang, Liqun Zhou, Yuanjie Niu, Ning Jiang

**Affiliations:** ^1^ Department of Urology, 2nd Hospital of Tianjin Medical University, Tianjin Institute of Urology, Tianjin 300211, China; ^2^ Department of Urology, Peking University First Hospital, The Institute of Urology, Peking University, Beijing 100034, China

**Keywords:** CK7, renal carcinomas, meta-analysis

## Abstract

Renal Oncocytomas and renal cell carcinomas (RCCs) share a common phenotype. This makes it very difficult to differentiate between the two tumors. Here, this study was to confirmed and expanded the findings that CK7 as a promising tool differentiate RCC from Oncocytomas across various geographic regions. A systematic search of databases was carried out and other relevant articles were also identified. Then the meta-analyses were conducted for 1,711 participants according to the standard guidelines. A total of 21 studies were included on the basis of inclusion criteria. CK7 by IHC was significantly associated with increased diagnosis of RCC (OR=10.64; 95% CI, 7.44-15.23; *P*=0.0001). Subgroup-analysis showed that findings didn't substantially change when only Caucasians or Asians (OR=10.58; 95% CI, 6.97-16.07; *P*<0.01 or OR=10.83; 95% CI, 5.39-21.74; *P*=0.004) were considered. There was also no significant publication bias observed. Our findings provide further evidences that the expression of CK7 contribute to differentiate RCC from Oncocytomas. CK7 protein overexpression was found in RCC, low expression in any of Oncocytomas. CK7 is potentially an important renal tumor marker.

## INTRODUCTION

Renal cell carcinomas (RCC) comprises 2-3% of all non-cutaneous malignant neoplasms in adults of both genders [1]. There are estimated 63,920 new cases and 13,860 deaths from renal cancer in the United States in 2014 [2]. Renal epithelial tumors arise from renal tubules and use to be classified into 4 major categories based on morphology, they are, clear cell renal carcinomas (ccRCCs) (75%), papillary renal carcinomas (PRCCs) (15%), chromophobe renal cell carcinoma (chRCC) (5%), and oncocytomas (5%) [3]. In the 2004, the World Health Organization (WHO) classified renal-tumor oncocytomas as benign neoplasms, the reported incidence rate of oncocytomas varies from 3.2% to 7%[4]. Accurate distinction between renal cell carcinomas and renal oncocytomas have significant prognostic.

CKs are a class of intermediate filaments that are the basic markers of epithelial differentiation[5]. They consist of at least 20 distinct molecules, the expression of which depends on cell type and differentiation position, making them useful in differential diagnosis of many epithelial tumors[5].

CK7 are increased expressed in a variety of RCC but show a more restricted expression in normal tissues or benign neoplasms [5–7]. CK7 was helpful in several diagnostic RCC [8], [9], and a useful marker in the differential diagnosis of epithelial tumors., evaluation of CK7 as new markers of differentiating RCC (ccRCCs, PRCCs and chRCC) from Oncocytomas is needed.

In an attempt to confirm the potential role of CK7 expression as a prognostic biomarker, we completed a meta-analysis of CK7 expression in patient of Asia and European lineage across different geographic regions with RCC and Oncocytomas.

### Meta-analysis results

When we pooled 21 eligible studies into the meta-analysis, result revealed that positive CK7 by IHC was significantly associated with increased diagnosis of RCC than Oncocytomas (OR=10.64; 95% CI, 7.44-15.23; *P*=0.0001) (Figure 2). Funnel plot asymmetry couldn't be observed (Figure 3), which suggested no evidence publication bias existing.

**Figure 1:**
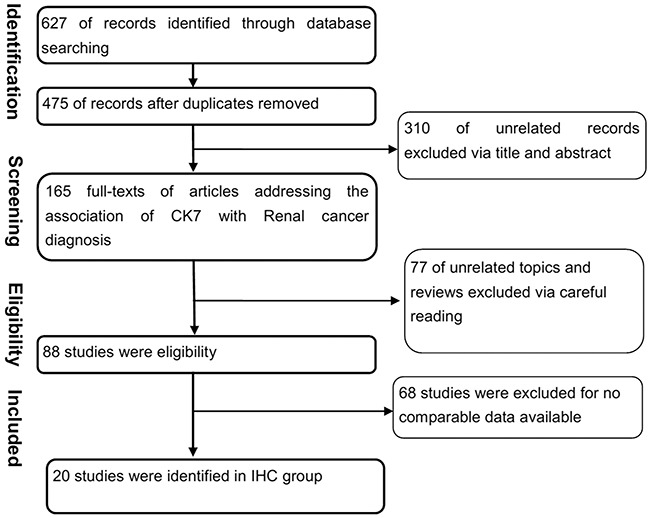
Flowchart of selecting process for meta-analysis A total of 628 articles were assembled. After full review, 21 articles were included.

**Figure 2:**
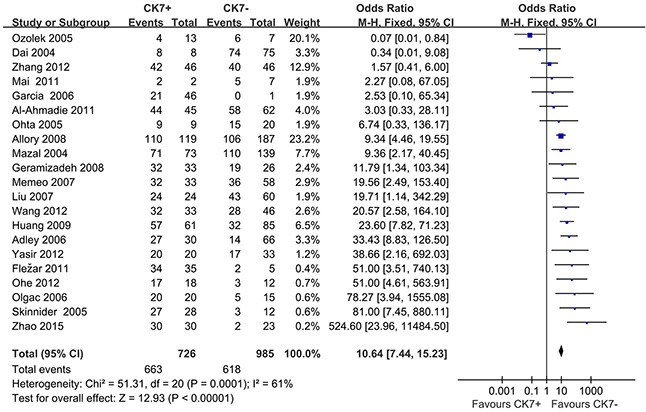
Forest plots for overall analysis of association of positive CK7 by immunohistochemistry with RCC and Oncocytomas, under random-effects model. M-H=Mantel-Haenszel method; CI=confidence interval

**Figure 3:**
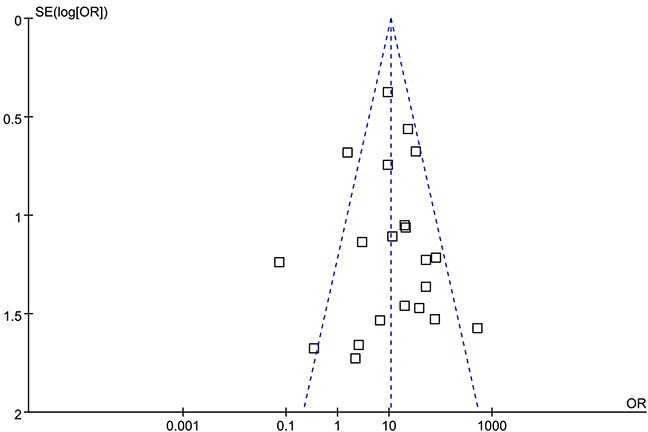
Funnel plots illustrating meta-analysis of overall analysis SE = standard error; OR = odds ratio.

**Figure 4:**
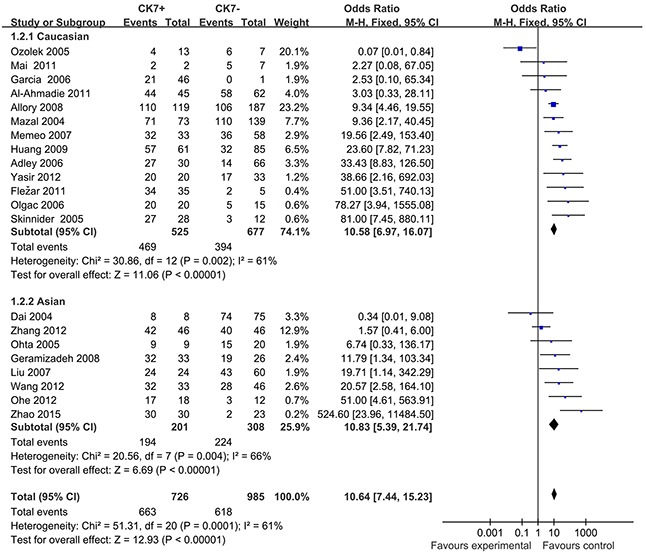
Forest plots for subgroup-analysis of association of positive CK7 by immunohistochemistry associated with RCC and Oncocytomas in Caucasians and Asians M-H=Mantel-Haenszel method; CI=confidence interval.

In consideration of the potential different expression of CK7 in different races, we yielded ethnicity-based subgroup-analyses (Figure 4). Subgroup-analysis showed that findings didn't substantially change when only Caucasians (OR=10.58; 95% CI, 6.97-16.07; *P*=0.002), or Asians were included (OR=10.83; 95% CI, 5.39-21.74; *P*=0.004). Both the results of subgroup-analyses showed that heterogeneity was usually a variation affecting the degree of risk rather than direction of effect.

## DISCUSSION

In this study, we explored the possible role of CK7 in distinguishing RCC from Renal Oncocytomas in 21 studies from various geographic regions including European and Asia[10–31]. CK7 expression by IHC was significantly associated with increased diagnosis of RCC (OR=10.64; 95% CI, 7.44-15.23; *P*=0.0001). The overall-analysis provided strong replication of the initial findings, confirming the CK7 for RCC.

All cases in our report followed the World Health Organization classification of renal tumors as standard level, based on a constellation of histologic features. It is difficult to make a correct histological diagnosis of RCC and Renal Oncocytomas based only on conventional routine staining, due to overlapping morphological characteristics[32–35]. Many researchers worked hard to find a way to differentiate RCC from Renal Oncocytomas. Some investigators have unsuccessfully reported that colloidal iron staining is not specific for distinguishing RCC from Oncocytomas[36,37]. But Ancillary methods, including histochemical and immunohistochemical stains, have been shown to be useful in the differential diagnosis of renal neoplasms. We summarized CK7 staining in the majority of RCC diffusely expressing membranous and Oncocytoma being typically negative or, at most, focally positive in scattered cells[12,14,18]. Matthewreported that CK7 is helper for diagnosis in circulating tumor cells (CTCs) of tissue of origin in breast cancer, prostate cancer and more expression in gastrointestinal, respiratory and gynecological malignancies[38,39,40]. Kinney proved that CK7 differentiate from Metanephric adenoma and papillary renal cell carcinoma[41]. Few researcher reported CK7 is more expression in Oncocytomas than in RCC[10,11]. In our approach we evaluated the potential diagnostic use of the expression of CK7 distinguishing RCC from Renal Oncocytomas in 21 studies(OR=10.64; 95% CI, 7.44-15.23; *P*=0.0001). The meta-analysis is a method that can solve the problem created by low statistical power in a single study to draw a more robust conclusion than the body of evidence. Our findings suggest that Ck7 may increase RCC diagnosis in the future.

Strengths of this study include its large sample size. Because of this, the geographic regions were distinguished in subgroup-analyses. However, our results are based on unadjusted estimates.

## CONCLUSION

Meta-analysis of the comprehensive literature revealed that the CK7 expression was strongly associated with RCC risk from various regions. CK7 is helpful in distinguishing RCC from Oncocytomas. There was no varying between Caucasian and Asia man.

### Evidence acquisition

#### Search strategy and selection criteria

We carried out a comprehensive literature review with search terms (Table 1). A comprehensive and systematic search through Medline, Web of Science and the Cochrane Library. The last quest was updated on May 25, 2015. When more than one studies with the same population were identified, only the most recent or complete one was included in this meta-analysis.

**Table 1:** Characteristics of trials included in meta-analyses

Study	Year	methods	Ethnicity	Cases		Controls		Study design	Control source
				Postive	total	Postive	total		
Memeo [14]	2007	IHC	Caucasian	32	68	1	23	cohort	Oncocytomas
Garcia [15]	2006	IHC	Caucasian	21	21	25	26	cohort	Oncocytomas
Skinnider [16]	2005	IHC	Caucasian	27	30	1	10	cohort	Oncocytomas
Al-Ahmadie [17]	2011	IHC	Caucasian	44	102	1	5	cohort	Oncocytomas
Huang [18]	2009	IHC	Caucasian	57	89	4	57	cohort	Oncocytomas
Mai [19]	2011	IHC	Caucasian	2	7	0	2	cohort	Oncocytomas
Yasir [20]	2012	IHC	Caucasian	20	37	0	16	cohort	Oncocytomas
Olgac [21]	2006	IHC	Caucasian	20	25	0	10	cohort	Oncocytomas
Allory [22]	2008	IHC	Caucasian	110	216	9	90	cohort	Oncocytomas
Fležar [23]	2011	IHC	Caucasian	34	36	1	4	cohort	Oncocytomas
Mazal [24]	2004	IHC	Caucasian	71	73	110	139	cohort	Oncocytomas
Skinnider [25]	2005	IHC	Caucasian	27	45	1	10	cohort	Oncocytomas
Ozolek [26]	2005	IHC	Caucasian	4	10	9	10	cohort	Oncocytomas
Adley [27]	2006	IHC	Caucasian	27	41	3	55	cohort	Oncocytomas
Liu [28]	2007	IHC	Asia	24	67	0	17	cohort	Oncocytomasl
Ohta [29]	2005	IHC	Asia	9	24	0	5	cohort	Oncocytomas
Ohe [30]	2012	IHC	Asia	17	20	1	10	cohort	Oncocytomas
Wang [31]	2012	IHC	Asia	32	60	1	19	cohort	Oncocytomas
Zhang Z [32]	2012	IHC	Asia	42	82	4	10	cohort	Oncocytomas
Dai [33]	2004	IHC	Asia	8	82	0	1	cohort	Oncocytomas
Zhao [30]	2015	IHC	Asia	30	32	0	21	cohort	Oncocytomas
Geramizadeh [31]	2008	IHC	Asia	32	51	1	8	cohort	Oncocytomas

IHC=Immunohistochemistry;

Studies were included if they fulfilled the following criteria: 1) studies that included the pathologically confirmed diagnosis of RCC, 2) the control group consisted of subjects who were the pathologically confirmed diagnosis of Oncocytomas, 3) studies that offered a hazard ratio (HR) and 95% confidence interval (CI) categorically or the data presented were available for calculation of the HR and 95% CI.

#### Data extraction and quality assessment

This meta-analysis was conducted according to the Preferred Reporting Items for Systematic Reviews and Meta-Analyses (PRISMA) [42] and Meta-analysis of Observational Studies in Epidemiology (MOOSE) [43] guidelines.

Study ethnicity of included subjects, numbers of cases and control subjects, and positive staining were extracted for factors of interest. The authors of published studies were also contacted for requesting necessary data that were not provided. Quality assessment was undertaken independently by at least four authors (Ning Jiang, Fuling Ma, Liang Dai, Zhun Wang). Two authors (Liqun Zhou, Yuanjie Niu) independently did the literature search and carefully extracted data. Any disagreements were resolved through discussion with authors (Niu and Jiang).

#### Data analysis and presentation

We used the crude odds radio (OR) with their corresponding 95 % confidence intervals (CI) as the metric of choice. The random effects model of DerSimonian and Laird was prespecified for use in all estimates because of the suspected a priori that studies were conducted by various authors with different populations and had different designs (eg, case-control and case series studies). Heterogeneity was evaluated using the Q test [44]. We also calculated the quantity *I*^2^ statistic that represented the percentage of total variation across studies. As a guide, (I^2^=0–25 %: no heterogeneity; I^2^=25–50 %: moderate heterogeneity; I^2^=50–75 %: large heterogeneity; I^2^=75–100 %: extreme heterogeneity) [45]. The funnel plot was addressed to reveal the potential publication bias. All analyses were conducted using Review Manage, version 5.2 (The Cochrane Collaboration, Oxford, U.K.).

### Evidence synthesis

#### Literature search and characteristics of studies

Initially, we assembled a total of 629 articles. After review of the abstracts, 166 studies were identified as potentially eligible for inclusion. After full review, 21 studies [10–31] using immunohistochemical method (IHC) were deemed eligible and were included in the study. The list of studies excluded and reasons for exclusion are shown in Figure 1.

The included studies were published from 2004 to 2015. Six conducted in Asia, the others in western countries. Most of included studies chose Oncocytomas. The details were listed in Table 1.
