# Correction: Evaluating prognosis by CK7 differentiating renal cell carcinomas from oncocytomas can be used as a promising tool for optimizing diagnosis strategies

**DOI:** 10.18632/oncotarget.25726

**Published:** 2018-06-26

**Authors:** Fuling Ma, Liang Dai, Zhun Wang, Liqun Zhou, Yuanjie Niu, Ning Jiang

**Affiliations:** ^1^ Department of Urology, 2nd Hospital of Tianjin Medical University, Tianjin Institute of Urology, Tianjin 300211, China; ^2^ Department of Urology, Peking University First Hospital, The Institute of Urology, Peking University, Beijing 100034, China

**This article has been corrected:** The complete funding information is given below:

**FUNDING**

The project was supported by a research grant of National Basic Research Program of China (973 Program, 2012CB518304). and the Science Foundation of Tianjin (No.: 11JCZDJC19700, 16JCZDJC34400) and 09ZCZDSF04300, 14KG135, 20140117 and the National Natural Science Foundation of China Grant numbers: 2012DFG32220 and 81572538, 81472682. Ph.D Programs Foundation of Ministry of Education of China 20131202110008.

Original article: Oncotarget. 2016; 7:46528-46535. https://doi.org/10.18632/oncotarget.10225

